# Accurate Diagnosis of COVID-19 by a Novel Immunogenic Secreted SARS-CoV-2 orf8 Protein

**DOI:** 10.1128/mBio.02431-20

**Published:** 2020-10-20

**Authors:** Xiaohui Wang, Joy-Yan Lam, Wan-Man Wong, Chun-Kit Yuen, Jian-Piao Cai, Shannon Wing-Ngor Au, Jasper Fuk-Woo Chan, Kelvin K. W. To, Kin-Hang Kok, Kwok-Yung Yuen

**Affiliations:** a Department of Microbiology, The University of Hong Kong, Hong Kong SAR, China; b State Key Laboratory for Emerging Infectious Diseases, The University of Hong Kong, Hong Kong SAR, China; c Carol Yu Centre for Infection, The University of Hong Kong, Hong Kong SAR, China; d Research Centre of Infection and Immunology, The University of Hong Kong, Hong Kong SAR, China; e School of Life Sciences, The Chinese University of Hong Kong, Hong Kong SAR, China; Virginia Polytechnic Institute and State University

**Keywords:** COVID-19, diagnosis, SARS-CoV-2, orf8

## Abstract

An accurate diagnostic test for early severe acute respiratory syndrome coronavirus 2 (SARS-CoV-2) infection is the key weapon to control the coronavirus disease 2019 (COVID-19) pandemic. We previously reported that the SARS-CoV-2 genome contains a unique orf8 accessory gene absent from other human-pathogenic coronaviruses. Here, we characterized the SARS-CoV-2 orf8 as a novel immunogenic secreted protein and utilized it for the accurate diagnosis of COVID-19. Extracellular orf8 protein was detected in cell culture supernatant and in sera of COVID-19 patients. In addition, orf8 was found highly immunogenic in COVID-19 patients, who showed early seropositivity for anti-orf8 IgM, IgG, and IgA. We hypothesize that orf8 secretion during SARS-CoV-2 infection facilitates early mounting of B cell response. The serological test detecting anti-orf8 IgG antibody can be used for the early and accurate diagnosis of COVID-19.

## Abstract (precis)

Current commercially available serological tests for COVID-19 patients are detecting antibodies against SARS-CoV-2 nucleoprotein and spike glycoprotein. The antinucleoprotein and antispike antibodies can be accurately detected in patients during the mid or late stage of infection, and therefore, these assays have not been widely used for early diagnosis of COVID-19. In this study, we characterized the secretory property of a SARS-CoV-2 orf8 protein and proposed that orf8 secretion during infection facilitates early mounting of the B cell response. We demonstrated the presence of anti-orf8 antibodies in both symptomatic and asymptomatic patients during the early stage of infection, while the anti-N antibody is not detected. Our serological test detecting anti-orf8 antibodies may facilitate the development of early and accurate diagnosis for COVID-19.

## INTRODUCTION

The novel severe acute respiratory syndrome coronavirus 2 (SARS-CoV-2) has already affected more than 17 million patients with more than half a million deaths within 7 months (1). One of the key epidemiological features of this novel coronavirus disease 2019 (COVID-19) is the presence of many asymptomatic patients with high viral loads which may contribute to its high transmissibility. Therefore, rapid and accurate diagnosis of early SARS-CoV-2 infection is important for the global control of COVID-19. Currently, molecular assays and serological assays are the two main recommended routine diagnostic tests. Molecular assays are based on reverse transcription-quantitative PCR (RT-qPCR). While they are sensitive and specific, the requirement of well-trained professionals and laboratory equipment forbids large-scale diagnostic screening in a community. Serological assays are rapid and inexpensive and can be performed at the point of care, but they are sometimes not suitable for early diagnosis.

Traditionally, serological tests may be used for the diagnosis of acute infection, confirmation of a positive molecular test result, retrospective determination of seroprevalence, and understanding of the immunological response. In general, host B cell response takes at least 3 to 5 days to produce antibodies after the first exposure to viral antigens. The seroconversion rate also depends on various factors, including the immunogenicity of viral antigens, the viral immunosuppressive property, and the competence of the host’s immune response. In the case of COVID-19, recent studies indicated that antibodies against SARS-CoV-2 nucleoprotein (N) and receptor-binding domain (RBD) of spike protein can be detected in some patient sera as early as 1 week after the onset of disease (2–4). There are several commercially available serological assays that can detect serum anti-N or anti-RBD antibodies in almost all RT-PCR-positive COVID-19 patients at ≥28 days post-symptom onset (10).

SARS-CoV-2 is a positive-stranded enveloped virus belonging to the subgenus *Sarbecovirus*, genus *Betacoronavirus*. Unlike other human highly pathogenic coronaviruses, the SARS-CoV-2 genome contains a unique open reading frame 8 (orf8) gene (6). In this study, we characterized the SARS-CoV-2 orf8 as a novel immunogenic secreted protein and utilized it for accurate diagnosis of COVID-19. Extracellular orf8 protein was detected in cell culture supernatant and in sera of COVID-19 patients. In addition, orf8 was found to be highly immunogenic in COVID-19 patients, who showed early seropositivity for anti-orf8 IgM, IgG, and IgA. Most importantly, our serological test detected anti-orf8 antibodies in all COVID-19 patients’ sera while the anti-N antibody could not be detected, but their respiratory specimens were RT-qPCR positive. This suggested that the production of orf8 antibodies is much earlier than that of anti-N antibodies. We therefore hypothesized that orf8 secretion during early SARS-CoV-2 infection may promote early B cell response against the viral antigen orf8. Our serological test detecting anti-orf8 antibodies may facilitate the development of early and accurate diagnosis for COVID-19.

## RESULTS

### *In silico* characterization of SARS-CoV-2 orf8 protein.

**FIG 1:**
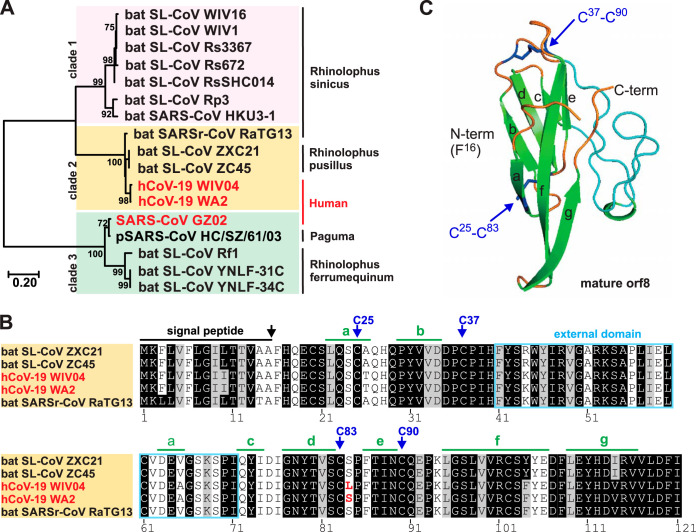
Characterization of SARS-CoV-2 orf8 protein. SARS-CoV-2 orf8 protein is unique in human-pathogenic coronaviruses. (A) Phylogenetic analysis of orf8 amino acid sequences. There are three clades of orf8 sequences originating from bat SARS-like coronaviruses (clade 1 [pink]), human SARS-CoV-2 and its closely related bat SARS-like coronaviruses (clade 2 [orange]), and human SARS-CoV and its closely related paguma and bat SARS-like coronaviruses (clade 3 [green]). (B) Multiple alignment of orf8 amino acid sequences. The signal peptide (black bar) is predicted in all clade 2 orf8 sequences. Four cysteine (C25, C37, C83, and C90) sites are predicted to form two disulfide bonds. The leucine (L)-to-serine (S) amino acid substitution at site 84 of SARS-CoV-2 orf8 is highlighted in red. Predicted strands (a to g) are highlighted (green bars). (C) *In silico* structural prediction of mature orf8 protein. The mature orf8 starts from the amino acid position 16 (N terminus) and end at the amino acid position 121 (C terminus). The predicted structure contains seven strands (wide green arrows) and loops (orange or blue lines) which are linked by two disulfide bonds. The unknown external domain is shown in blue.

The orf8 protein is absent in all human-pathogenic coronaviruses except the recent SARS-CoV-2, but it is present in some bat coronaviruses. We first examined the amino acid sequences of human SARS-CoV-2 orf8 and other orf8 proteins. The orf8 amino acid sequences of known bat SARS-CoV-like coronaviruses, recently discovered bat SARS-CoV-2-related coronaviruses, SARS-CoV and SARS-CoV-2 were segregated into three clades in the phylogenetic tree (Fig. 1; see also Fig. S1 in the supplemental material). Amino acid sequences of human SARS-CoV-2 orf8 are highly conserved and similar to that of clade 2 bat SARS-like coronaviruses (RaTG13, ZXC21, and ZC45) but less similar to clade 1 coronaviruses that include human SARS-CoV (GZ02), paguma SARS-CoV (HC/SZ/61/03) and bat SARS-like coronaviruses (WIV16, WIV1, Rs3367, Rs672, Rs SHC014, Rp3, and HKU3-1). Human SARS-CoV is known to contain a full-length orf8 when this zoonotic virus was first transmitted to humans, but it quickly split into orf8a and orf8b with 29-nucleotide deletion and remained the same throughout the rest of the time of the outbreak (7). In contrast, human SARS-CoV-2 orf8 (hCoV-19 HKU-SZ-005b [8]) is a relatively stable open reading frame that has only three amino acids that are different (I10L, F104Y, V114I) compared to its closest bat SARS-like coronaviruses (clade 2: ZXC21 and ZC45) (Fig. 1B). Taken together, orf8 is a unique protein in human SARS-CoV-2 and clade 2 bat SARS-like coronaviruses. The immunological response against orf8 in COVID-19 patients should be highly specific.

10.1128/mBio.02431-20.1 FIG S1 Multiple alignment of orf8 amino acid sequences. Amino acid sequences of orf8 proteins are derived from bat SARS-like coronaviruses (bat SL-CoV WIV16, GenBank accession no. KT444582; bat SL-CoV WIV1, KF367457; bat SL-CoV Rs3367, KC881006; bat SL-CoV Rs672, FJ588686; bat SL-CoV RsSHC014, KC881005; bat SL-CoV Rp3, DQ071615; bat SARS-CoV HKU3-1, DQ022305; bat SARSr-CoV RaTG13, MN996532; bat SL-CoV ZXC21, MG772934; bat SL-CoV ZC45, MG772933; bat SL-CoV Rf1, DQ412042; bat SL-CoV YNLF-31C, KP886808; bat SL-CoV YNLF34C, KP886809), Paguma SARS-CoV (pSARS-CoV HC/SZ/61/03, AY515512), SARS-CoV (SARS-CoV GZ02, AY390556), and SARS-CoV-2 (hCoV-19 WIV04, EPI_ISL_402124; hCoV-19 WA2, PI_ISL_412970). Multiple alignment analysis was performed, and the alignment of orf8 protein sequences was presented as a BoxShade diagram. There are three clades representing (i) bat SARS-like coronaviruses (highlighted in pink; clade 1), (ii) human SARS-CoV-2 and its closest bat coronaviruses (highlighted in orange; clade 2) and (iii) human SARS-CoV and its related paguma and bat coronaviruses (highlighted in green; clade 3). Black and gray boxes of amino acid letters represent identity and similarity, respectively. Download FIG S1, EPS file, 2.6 MB. Copyright © 2020 Wang et al. 2020 Wang et al. This content is distributed under the terms of the Creative Commons Attribution 4.0 International license.

We previously reported that SARS-CoV-2 orf8 is a putative secreted protein with an N-terminal signal peptide (6). The signal peptide is conserved between SARS-CoV-2 and the related bat coronaviruses (Fig. 1B). Using web-based bioinformatic tools, we here predicted the secondary structure of SARS-CoV-2 orf8 and found one short α-helix (α), seven β-strands (a to g) plus an unknown external domain (Fig. 1B). Four cysteine sites at 25, 37, 83, and 90 are predicted to form two disulfide bonds, which is consistent with other studies (7, 9). The tertiary structure of SARS-CoV-2 orf8 was further predicted using ROSETTA algorithms, of which comparative model of protein domains were calculated by HHSEARCH, SPARKS, and Raptor (Fig. 1C and Fig. S2). The predicted mature SARS-CoV-2 orf8 protein structure contains a core domain comprising seven α-helixes with two disulfide bonds, and an external domain (amino acid positions 51 to 71) that is absent in the clade 3 orf8 sequences (Fig. S1). The multiple alignment and *in silico* structural analysis suggested that SARS-CoV-2 orf8 is a novel viral protein, which is different from 2003 SARS-CoV orf8, orf8a, and orf8b proteins.

10.1128/mBio.02431-20.2 FIG S2 *In silico* structural prediction of mature orf8 protein. (A to C) Three-dimensional structure at 0° and 270° and top view of the mature orf8 (without signal peptide) is shown. β-Strands (green), predicted disulfide bonds (blue), amino acid loop (orange), and the external domain (light blue) are shown. (D) Overall topology of the predicted structure. Download FIG S2, EPS file, 2.5 MB. Copyright © 2020 Wang et al. 2020 Wang et al. This content is distributed under the terms of the Creative Commons Attribution 4.0 International license.

### SARS-CoV-2 orf8 is a secreted protein.

**FIG 2:**
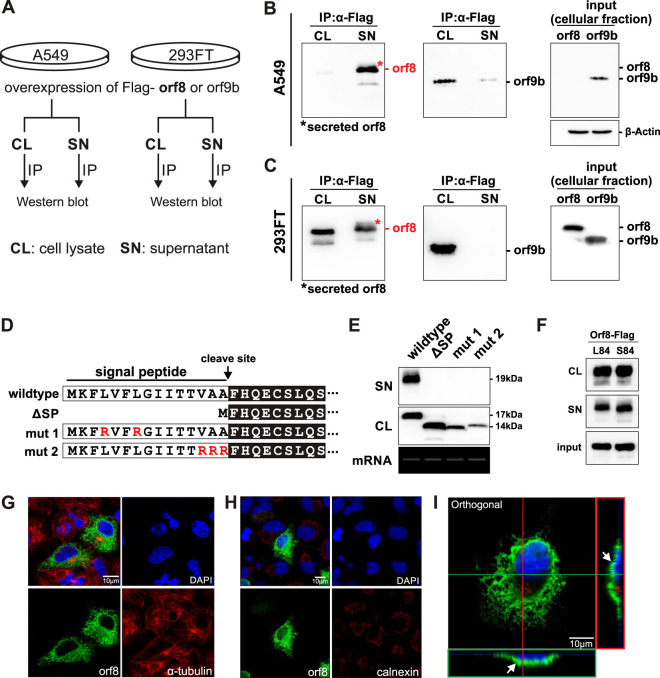
SARS-CoV-2 orf8 is actively secreted into culture supernatant. (A) Schematic diagram illustrating the method of orf8 detection in cell and culture supernatant. Plasmid DNA encoding the SARS-CoV-2 orf8 or orf9b with Flag tag was transfected into A549 and 293FT cells. At 48 h posttransfection, cell lysate (CL) and culture supernatant (SN) were harvested and orf8/orf9b proteins were immunoprecipitated (IP) and detected by Western blotting. (B and C) Orf8 is actively secreted into culture supernatant of A549 (B) and 293FT (C) cells. Secreted orf8 protein detected in culture supernatant is indicated by a red asterisk. α-Flag, anti-Flag antibody. (D) Diagram illustrating the N-terminal signal peptide mutants of orf8 used for the study of protein secretion. Three mutants include the following: (i) a signal peptide deletion mutant (ΔSP), (ii) mutation of internal hydrophobic leucine into hydrophilic arginine (mut 1), and (iii) mutation of three small uncharged amino acids near the proposed cleave sites into arginine (mut 2). (E) Plasmid DNA expressing Flag-tagged orf8 wild-type and signal peptide mutants were transfected into 293FT cells for 48 h. Intracellular orf8 (expressed in CL) and extracellular orf8 (secreted in SN) were detected as described above for panels B and C. Cellular expression of orf8 transcripts (mRNA) were detected by RT-PCR. (F) Intracellular and extracellular expression of two genotypes of orf8 were detected as described above for panels B and C. (G and H) Subcellular localization of orf8 protein. A549 cells were transfected with Flag-tagged orf8 and stained with anti-Flag (green in panels G and H), anti-α-tubulin (red in panel G), or anti-calnexin antibodies (red in panel H). The nucleus was stained and shown in blue color. (I) Z-stack visualization of A549 cells transfected with Flag-tagged orf8. Green signal represents Flag-tagged orf8, red signal represents GM130, and blue signal represents 4′,6′-diamidino-2-phenylindole (DAPI).

To characterize the novel SARS-CoV-2 orf8 protein, a basic overexpression model in mammalian cell lines was adopted to preliminarily test its function. In two human cell lines, lung epithelial A549 cells and embryonic kidney 293FT cells, ectopic expression of orf8 did not cause cell death or morphological changes (Fig. S3). On the basis of the sequence and structural analyses, we predicted that the observed signal sequence at the N terminus of orf8 may be responsible for extracellular secretion of protein. To test this hypothesis, C-terminal Flag-tagged orf8 was overexpressed in 293FT and A549 cells. The intracellular and extracellular orf8 protein was immunoprecipitated by anti-Flag beads and detected by Western blotting (Fig. 2A). Consistent with our hypothesis, abundant orf8 protein, but not the control orf9b protein of similar size, could be detected in the culture supernatant of both cell lines, confirming that SARS-CoV-2 orf8 is a secreted protein (Fig. 2B and C).

10.1128/mBio.02431-20.3 FIG S3 No morphological changes were observed in orf8-expressing A549 cells. A549 cells were transfected with empty vector or Flag-orf8 expression vector as described in the legend to Fig. 2G to I. At 48 h posttransfection, bright-field images were captured using a Nikon Eclipse Ti microscope. Download FIG S3, EPS file, 2.5 MB. Copyright © 2020 Wang et al. 2020 Wang et al. This content is distributed under the terms of the Creative Commons Attribution 4.0 International license.

We further characterized the secretion property of the orf8 protein by introducing mutations to the signal sequence (Fig. 2D). Three mutants were generated: deletion of the signal peptide (ΔSP), mutation of two internal hydrophobic leucine to hydrophilic arginine (mut 1), and mutation of three small uncharged amino acids near the predicted cleavage site to arginine (mut 2). The wild-type orf8 protein and the three orf8 mutants were expressed in 293FT cells, and the presence of intracellular and extracellular orf8 protein was detected as previously described. Strikingly, extracellular orf8 could not be detected in all three mutants (Fig. 2E), indicating that the signal peptide is essential for extracellular secretion of orf8. It should also be noted that there is a 3-kDa size difference between wild-type and mutant orf8, which might suggest that the signal peptide could be important for proper posttranslational modification of orf8. There are currently two genotypes (L84 and S84) found in circulating SARS-CoV-2. However, this polymorphism does not affect secretion or stability of orf8, as orf8 protein of both genotypes could be found in supernatant of transfected cells (Fig. 2F). In addition, we examined the intracellular localization of orf8 protein in A549 cells (Fig. 2G to I). Interestingly, orf8 did not localize to the endoplasmic reticulum (ER) (Fig. 2H). Instead, it concentrated near the cell surface when we examined its three-dimensional (3D) localization, which is in line with the secretory nature of the orf8 protein (Fig. 2I; see also Movie S1 in the supplemental material).

10.1128/mBio.02431-20.5 MOVIE S1 3D localization orf8 protein in lung A549 epithelial cells. Video illustration of Flag-orf8 staining in A549 cells. This 3D movie was exported from the same confocal field as illustrated in Fig. 2I. “Blanket”-like staining pattern of orf8 is visualized at the cell surface. Download Movie S1, AVI file, 8.0 MB. Copyright © 2020 Wang et al. 2020 Wang et al. This content is distributed under the terms of the Creative Commons Attribution 4.0 International license.

### SARS-CoV-2 orf8 peptides found in sera of COVID-19 patients.

**FIG 3:**
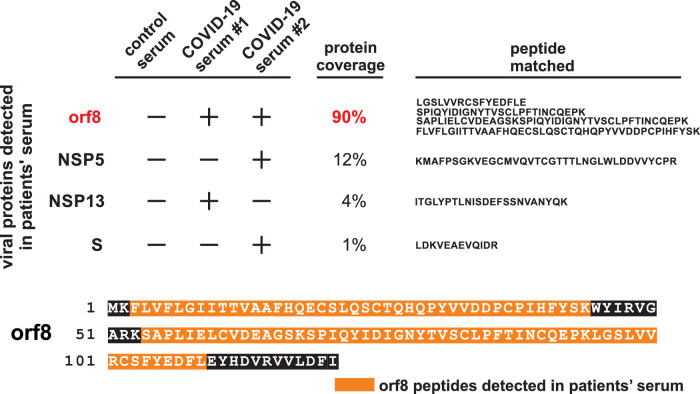
SARS-CoV-2 orf8 peptides found in the sera of COVID-19 patients. The orf8 peptide was abundantly detected in SARS-CoV-2-infected patient serum. Serum samples were subjected to liquid chromatography-mass spectrometry analysis. Sequence coverage of SARS-CoV-2 orf8 in the serum samples of two COVID-19 patients are shown. The peptides identified in both samples are highlighted in orange.

To validate the secretion of orf8 protein during infection and its clinical relevance, sera of COVID-19 patients post-symptom onset were analyzed for the presence of the orf8 protein. First, 14 of the most abundant serum proteins, including serum albumin and immunoglobulins, were removed. The presence of SARS-CoV-2 proteins in the depleted sera was then determined by liquid chromatography-mass spectrometry. Impressively, four unique peptides covering 90% of the orf8 amino acid sequence could be detected despite the small size (13 kDa) of orf8 (Fig. 3). We also detected three viral peptides corresponding to spike, nonstructural protein 5 (nsp5) and nsp13, although the protein coverage and unique peptide count were much lower. Since anti-orf8 antibody is not yet available, the exact abundance of serum orf8 protein could not be accurately quantitated at this moment. However, the exceptionally high protein coverage suggested that abundant orf8 protein is secreted by infected cells. It is therefore intriguing to understand any possible interactions between the host immune response and this ubiquitous orf8 protein present in the serum and its clinical significance.

### The orf8 protein is highly immunogenic in COVID-19 patients.

**FIG 4:**
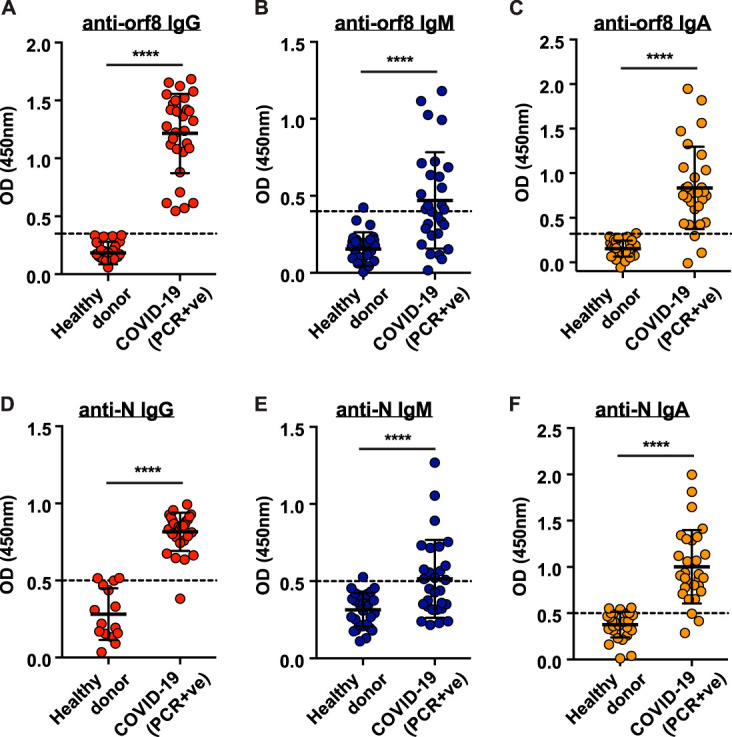
Abundant anti-orf8 antibodies detected in the sera of COVID-19 patients ≥28 days post-symptom onset. (A to C) Titers of antibodies against SARS-CoV2 orf8 in 29 hospitalized patients’ sera collected ≥28 days post-symptom onset and healthy control donors (*n* = 30) were examined by ELISA. OD, optical density; PCR+ve, PCR positive. (D to F) Serum titers of antibodies against SARS-CoV-2 nucleoprotein (N) in the patients and healthy blood donors (A to C) were examined by ELISA. Serum samples were tested at a dilution of 1:100. *P* values were calculated by Mann-Whitney U test. The dashed lines represent cutoff values (the mean absorbance at 450 nm of serum samples obtained from healthy control donors plus two times the standard deviation). Data were presented as means ± standard deviations (SD) (error bars). ****, *P* < 0.0001.

Currently, there is insufficient knowledge as to when and what kind of immune responses were elicited by orf8. Since orf8 is a secreted viral protein that could be detected in sera of hospitalized COVID-19 patients, we questioned whether orf8 is immunogenic. Therefore, we tested for the presence of anti-orf8 antibodies in infected patients using our in-house orf8 enzyme-linked immunosorbent assay (ELISA). Serum samples were collected from 29 adult COVID-19 patients ≥28 days post-symptom onset (median, 46 days; range, 28 to 62 days) (see Table S1 in the supplemental material). All patients were confirmed to have COVID-19 by RT-qPCR during their acute phase of infection. Compared to pre-epidemic control sera collected in 2019 from healthy donors, all tested COVID-19 patient serum samples were positive for IgG against orf8 in our in-house orf8 ELISA (Fig. 4A). Moreover, the seropositive rates for anti-orf8 IgM (51.7%, 15/29) and IgA (89.7%, 26/29) were also fairly high (Fig. 4B and C). Our previous work revealed that the titers of anti-N IgG, IgM, and IgA in COVID-19 patients increases over time following infection (4). We therefore also analyzed the abundance of anti-N antibodies by our in-house N ELISA. In line with our previous finding, the seropositive rates regarding anti-N IgG (96.6%, 28/29), IgM (48.3%, 14/29), and IgA (89.7%, 26/29) (Fig. 4D to F) were reasonably high in this cohort of patients. These results suggest that orf8 is highly immunogenic in eliciting antibody response in COVID-19 patients.

10.1128/mBio.02431-20.4 TABLE S1 Cohort characteristics of patients. Characteristics of patients involved in this study were summarized in the table. Download Table S1, DOCX file, 0.02 MB. Copyright © 2020 Wang et al. 2020 Wang et al. This content is distributed under the terms of the Creative Commons Attribution 4.0 International license.

### Early and accurate diagnosis by anti-orf8 IgG ELISA.

**FIG 5:**
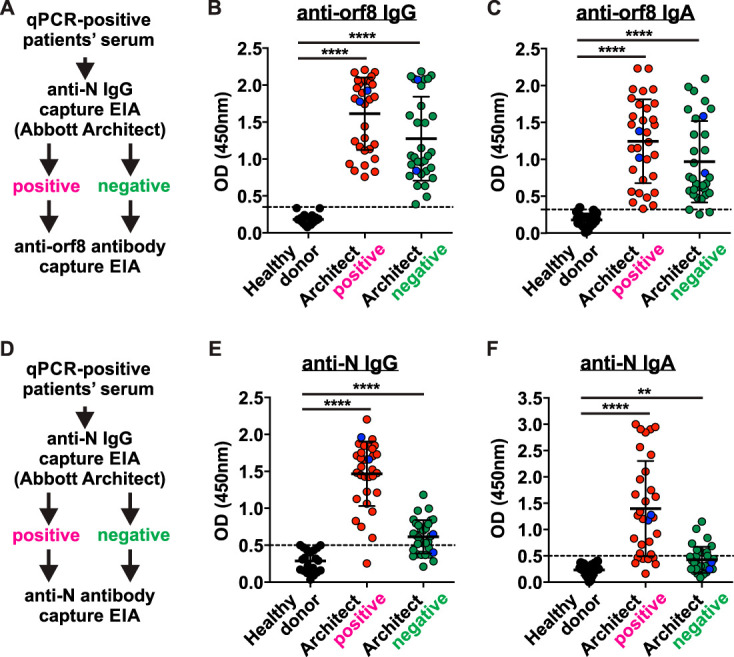
Detection of anti-orf8 IgG in anti-N IgG-negative serum samples from COVID-19 patients. Anti-orf8 antibodies are detectable in RT-qPCR-confirmed but Abbot Architect seronegative COVID-19 cases. (A to C) orf8-specific IgG and IgA in 64 serum samples from PCR-confirmed COVID-19 cases and healthy control donors (*n* = 20) were quantified by ELISA. The patient sera were divided into two groups: seropositive (*n* = 32) and seronegative (*n* = 32) based on the results of Abbott Architect SARS-CoV-2 N IgG assay. EIA, enzyme immunoassay. (D to F) SARS-CoV-2 N-specific IgG and IgA in serum samples in panels A and B were examined using in-house N ELISA. The dashed lines represent cutoff values. Data were presented as means ± SD. **, *P* < 0.01; ****, *P* < 0.0001.

There are several commercially available serological assays that can detect anti-N or anti-RBD antibodies in almost all PCR-positive serum samples from COVID-19 patients at ≥28 days post-symptom onset (10). However, the percentage of seropositivity goes down when the sera were harvested at early time points, i.e., less than 28 days post-symptom onset, which renders such tests not reliable for early diagnosis and management. As the secreted orf8 protein can efficiently elicit anti-orf8 antibody response in infected patients (Fig. 4), we asked a key question of whether anti-orf8 antibodies could be detected during early infection when anti-N/RBD is not yet detected. We therefore further examined the presence of anti-orf8 antibodies in a panel of 64 sera from RT-qPCR-confirmed COVID-19 patients at ≤28 days post-symptom onset (median, 4 days; range, 0 to 14 days) (Fig. 5A). This panel comprises 32 anti-N seropositive samples (Abbott positive) and 32 anti-N seronegative samples (Abbott negative) as diagnosed by automated Abbott Architect SARS-CoV-2 IgG immunoassay. Surprisingly, anti-orf8 IgG antibodies could be detected in all 64 serum samples, including the 32 Abbot-negative samples (Fig. 5B). In addition, we examined the presence of anti-orf8 IgA antibodies in both Abbott-positive and -negative serum samples and found that 95.3% (61/64) were anti-orf8 IgA positive (Fig. 5C). Notably, 4 out of the 64 PCR-confirmed patients were asymptomatic but showed seropositivity for both anti-orf8 IgG and IgA (blue dots in Fig. 5B and C). Next, we profiled anti-N IgG and IgA levels using our in-house anti-N ELISA (4, 11) (Fig. 5D). Consistent with the diagnostic result of Abbott Architect SARS-CoV-2 IgG immunoassay, the Abbott-seropositive samples yielded significantly increased anti-N IgG and IgA compared with healthy donors, while the Abbott-negative samples constituted around 68.8% or 39.1% of the readings above the cutoff lines (Fig. 5E and F). These results suggest that detection of anti-orf8 antibodies is more sensitive and accurate than the conventional anti-N antibody detection in identifying COVID-19 patients especially during the early stage of infection.

### Successful detection of anti-orf8 IgG in serial patient sera during early hospitalization.

**FIG 6:**
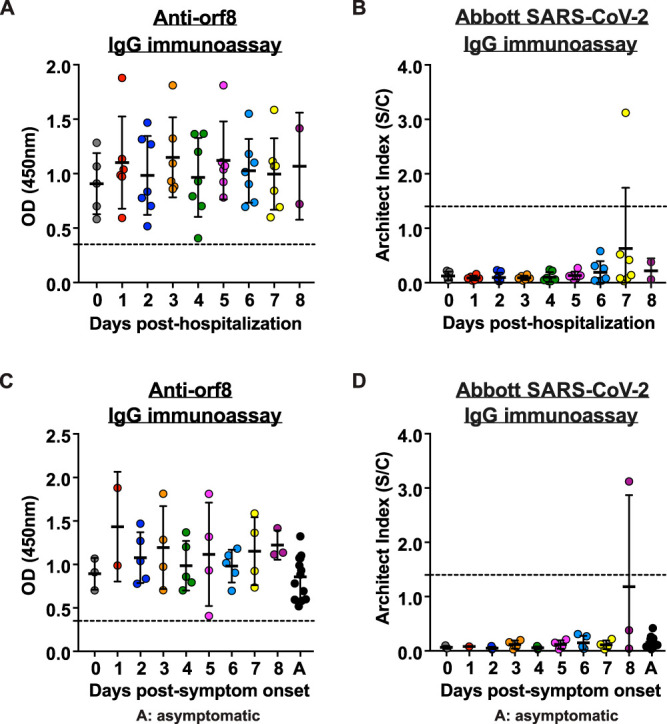
Detection of serum anti-orf8 IgG during early hospitalization of COVID-19 patients. Detection of anti-orf8 antibodies in RT-qPCR confirmed symptomatic and asymptomatic COVID-19 patients. (A) Anti-orf8 IgG was quantitated in serial serum samples obtained from 14 RT-qPCR-confirmed COVID-19 patients. The dashed lines represent cutoff values. (B) Anti-N IgG was quantitated by Abbott Architect SARS-Cov-2 IgG assay in the same set of serum samples tested in panel A. (C and D) Data generated from panels A and B were represented as days post-symptom onset for 10 symptomatic and 4 asymptomatic COVID-19 patients. Data were statistically analyzed and presented as means ± SD.

Due to the limitation that anti-N/RBD antibodies are generally detected only at the mid or late stage of acute infection, for example, more than 10 days after disease onset (2–4), current serological tests detecting anti-N/RBD antibodies can be used only for confirmation of molecular test results, determination of seroprevalence, and understanding the immunological responses, but not for the early diagnosis and management of acute COVID-19. However, we demonstrated that anti-orf8 IgG antibodies can be detected in all 64 sera from RT-qPCR-confirmed COVID-19 patients at ≤28 days post-symptom onset, irrespective of their anti-N status (Fig. 5B). This finding opens the possibility that anti-orf8 ELISA may be suitable for the diagnosis for acute infection. Therefore, we further examined serial serum samples of 14 RT-qPCR-confirmed COVID-19 patients. Serum samples were collected from day 0 (the first day of hospitalization) to day 8 (median, 4 days; range, 0 to 8) posthospitalization. All of these serum samples were anti-orf8 IgG positive on day 0 and persistently positive throughout the monitored period (Fig. 6A). As expected, anti-N antibody was mostly not detected by the Abbott Architect SARS-CoV-2 IgG assay (Fig. 6B). A key determinant to control the COVID-19 pandemic is whether we can identify infected people who do not show or have not yet shown disease symptoms. We therefore further represented the same set of data using the symptom onset date as reference (Fig. 6C and D). Surprisingly, anti-orf8 IgG antibodies could already be detected on the day of symptom onset in all patients. Moreover, all sera from four asymptomatic patients were also anti-orf8 seropositive (black dots in Fig. 6C). Taken together, this novel immunogenic secreted orf8 protein can be used as an antigen for accurate and early serological diagnosis of COVID-19.

## DISCUSSION

Current studies have reported that COVID-19 patients develop neutralizing antibodies and high titer of SARS-CoV-2-specific antibody response (4, 12, 13). The assays against the antibodies targeting spike and N proteins have been utilizing effective diagnostic strategies such as ELISA, chemoluminescence assays (CLIA) and lateral flow assays (LFA) (12). However, the major limitation of the detection of antibodies against spike and N proteins is that they are negative in the acute phase of infection, rendering a lower sensitivity of antibody-mediated diagnosis (13). It is noteworthy that 10 out of 175 confirmed patients did not develop a neutralizing SARS-CoV-2-specific antibody response examined by pseudoparticle assay, whereas binding antibodies were still detected by ELISA (13). In one recent report on 37 asymptomatic and 37 symptomatic patients, more than 90% of both groups showed steep declines in the levels of N and spike protein-specific IgG antibodies within 2 to 3 months after infection (14). Similarly, there are reports of decreased spike protein-specific and neutralizing antibodies in convalescent patients with COVID-19 (13). These instances of no or low antibody responses against N and spike protein may lead to an underestimation of early or asymptomatic infections and threaten the success of a potential vaccine that targets the spike protein alone. Therefore, identification of additional viral antigens with high immunogenicity and specificity in both symptomatic and asymptomatic individuals are urgently needed for timely serosurveillance and vaccine development.

SARS-CoV-2 orf8 is unique protein that is absent in other human-pathogenic coronaviruses. Here, we demonstrated that orf8 is a secreted protein (Fig. 2) which can be detected in serum samples of COVID-19 patients (Fig. 3). Compared to the antibody response against SARS-CoV-2 N, orf8 elicits expedited seroconversion during early SARS-CoV-2 infection (Fig. 5 and 6). Our *in vitro* orf8 secretion assay suggested that orf8 could be readily secreted upon expression (Fig. 2). We therefore hypothesized that the secreted orf8 during the early infection, perhaps within the first 48 h, might have elicited an early B cell response to produce anti-orf8 antibodies before or right after symptom onset, leading to the detection of anti-orf8 antibodies during very early stage of infection after symptom onset that we observed (Fig. 6). Most importantly, anti-orf8 antibodies, but not anti-N antibodies, can be detected in asymptomatic patients (Fig. 5 and 6). Our results suggested that the detection of antibodies against this novel immunogenic secreted orf8 protein can be applied for accurate and early diagnosis of COVID-19 disease. This is not unexpected because immunological profiles of our COVID-19 patients indicate that B cell response is preserved despite broad impairment of T, NK, monocyte and dendritic cell functions and decrease in cell count during the acute phase of infection (15). Further development of inexpensive anti-SARS-CoV-2 orf8 IgG/IgA cassette rapid test will greatly facilitate early diagnosis at the point of care or even large-scale screening in the community, where dedicated laboratory apparatus and professionals are not readily available.

As SARS-CoV-2 is a newly emerging virus circulating in humans, frequent mutations were reported in circulating SARS-CoV-2. A survey with samples collected from January 2020 to March 2020 described a 382-nucleotide deletion (Δ382) in a SARS-CoV-2 genome in Singapore (45/191 screened samples) and Taiwan (one case) (16, 17). This deletion truncates open reading frame 7b (ORF7b) and ORF8, removing the orf8 transcription regulatory sequence (TRS) and eliminating orf8 transcription. Moreover, the Δ382 mutant showed significantly higher replicative fitness *in vitro* than the wild-type strain, while no difference was observed in viral replication (17). However, as the Δ382 mutant is no longer circulating in Singapore which is likely due to the aggressive surveillance and quarantine. Moreover, mutations that cause deletion of orf8 have not been reported in other regions of the world. This is unclear whether such a mutation is still evolving and facilitating its adaptation to human immune system. For the patients infected with orf8-deleted mutants, a parallel detection with other diagnostic methods such as RT-PCR is still required.

This study did not investigate cellular immunity to COVID-19, i.e., immune response involving T cells rather than antibodies. Some previous studies have shown the necessity of sufficient magnitude or durability of T cell response for antibody generation (18–21). A study of 41 recovered COVID-19 patients found consistent detection of neutralizing antibodies, memory B cells and circulating follicular helper T cells against spike protein (18). It remains unclear whether a robust B cell response against orf8 correlates with an early and long-lasting T cell response. Further investigations on orf8 protein and epitopes recognized by both human B and T cell responses is of immediate relevance for assisting candidate vaccine design and facilitating evaluation of vaccine candidate immunogenicity.

Although the anti-orf8 antibodies may aid in the diagnosis of COVID-19 disease, there is also great uncertainty about whether adaptive immune responses to orf8 are protective or pathogenic and whether this might influence susceptibility to COVID-19 disease. For example, the possibility that anti-orf8 antibodies contribute to host pathogenesis by forming immune complex with orf8 protein should be evaluated in future studies. Additionally, to date, the duration of SARS-CoV-2 RNA shedding has not been well characterized. One recent study of 191 COVID-19 patients reported that the median duration of viral shedding was 20 days in survivors (range, 8 to 37 days) (22). In this study, we observed anti-orf8 IgG levels in all the RT-PCR-confirmed SARS-CoV-2-infected individuals upon and within 2 months of the symptom onset (Fig. 4). Notably, the anti-orf8 IgG antibodies are also detected in asymptomatic patients (Fig. 5 and 6). The durability of the antibodies against orf8 and its correlation with host pathogenesis in the short and long term needs to be determined through serological testing of the convalescent COVID-19 patients.

The function of extracellular or secreted orf8 protein has not yet been reported. Some of the secreted viral proteins function as cytokine mimics (e.g., Epstein-Barr virus [EBV] viral interleukin 10 [vIL-10]), cytokine inhibitors (e.g., herpesvirus M3), complement inhibitors (e.g., vaccinia virus complement control protein [VCP]) and inflammatory cell inhibitor (myxoma virus SERP-I) (23). It will be of great interest to further investigate the function of secreted orf8 during infection.

In summary, we provide experimental evidence that orf8 functions as a secreted protein that is highly immunogenic. To the best of our knowledge, this study is the first to report the secretory property of SARS-CoV-2 orf8 and the earlier induction and higher sensitivity of anti-orf8 antibodies compared to that of anti-N antibodies. Our findings suggest the ELISA described in this study can be directly applied for the early and accurate diagnosis of COVID-19.

## MATERIALS AND METHODS

### Patient cohorts.

COVID-19 patients with respiratory samples positive for SARS-CoV-2 by reverse transcription-quantitative PCR (RT-qPCR) after admission to the Queen Mary Hospital in Hong Kong during January and July 2021 were included in this study. An in-house RT-qPCR targeting the SARS-CoV-2 RNA-dependent RNA polymerase-helicase gene region was performed as we previously described (4, 11). In Hong Kong, patients were tested for SARS-CoV-2 based on clinical and epidemiological criteria as outlined and updated by the Hospital Authority. Final confirmation of initially positive specimens using nasopharyngeal or sputum specimens was done at the Public Health Laboratory Centre of Hong Kong. The negative-control sera were from Hong Kong blood donors collected in October 2019 (before the emergence of COVID-19). This study was approved by the Institutional Review Board of the University of Hong Kong/Hospital Authority Hong Kong West Cluster (UW 13-372). Since archived specimens were used, written informed consent was waived.

### Plasmids.

Coding sequences of C-terminus Flag-tagged or His-tagged orf8 were subcloned into mammalian expression vector pCAGEN (Addgene plasmid no. 11160). Gene fragments for signal peptide mutation and L84S amino acid substitution were generated by PCR and subcloned into the same pCAGEN backbone vector.

### Cell culture.

Human embryonic kidney 293FT cells were purchased from Invitrogen (USA), and human lung epithelial A549 cells were obtained from ATCC (USA) (ATCC CCL-185). Both cell lines were cultured in Dulbecco’s modified Eagle medium (Thermo Fisher Scientific, USA) with 10% fetal bovine serum (DMEM/10% FBS) and incubated at 37°C with 5% CO_2_. Expi293F cells were purchased from Thermo Fisher Scientific and used for the protein production. Suspension Expi293F cells were cultured in serum-free and protein-free Expi293 expression medium (Thermo Fisher Scientific) and incubated at 37°C with 8% CO_2_ and shaking at 125 rpm.

### Transfections.

For 293FT, Flag-tagged orf8 expression plasmid was transfected using GeneJuice transfection reagent (Millipore, USA) according to the manufacturer’s instructions. Cells or culture supernatant was harvested according to experiment conditions. For A549, Flag-tagged orf8 expression plasmid was transfected using Lipofectamine 3000 transfection reagent (Thermo Fisher Scientific) according to the manufacturer’s instructions. At 5 h posttransfection, cells were washed with phosphate-buffered saline (PBS) two times and replenished with fresh DMEM/10% FBS. Cells or culture supernatant was harvested at the indicated time points.

### Recombinant SARS-CoV-2 orf8 protein production and purification.

Recombinant orf8 is highly insoluble and cannot be expressed in Escherichia coli. Therefore, the Expi293 expression system (Thermo Fisher Scientific) was used for mammalian recombinant orf8 protein production. Expi293F cells were transfected with pCAGEN-orf8-His using Expifectamine 293 (Thermo Fisher Scientific) according to the manufacturer’s instructions. Five or 6 days posttransfection, serum-free medium carrying secreted orf8 protein was harvested and purified by nickel-nitrilotriacetic acid (Ni-NTA) agarose (Qiagen, USA) or anti-His affinity resin (Genscript). Recombinant His-orf8 protein was eluted using 200 mM imidazole in 500 mM NaCl or eluted with low pH buffer.

### Western blotting.

Transfected cells were harvested and lysed with radioimmunoprecipitation assay (RIPA) buffer (50 mM Tris-Cl [pH 8.0], 150 mM NaCl, 1% NP-40, 0.1% sodium dodecyl sulfate [SDS], 0.5% sodium deoxycholate) with EDTA-free protease inhibitor cocktail (Roche). Clarified protein lysates were mixed with 5× protein sample buffer (10% SDS, 25% 2-mercaptoethanol, 50% glycerol, 0.01% bromophenol blue, 300 mM Tris-Cl [pH 6.8]) and denatured at 95°C. Proteins were then separated by 12% SDS-polyacrylamide gel electrophoresis (PAGE), transferred onto polyvinylidene fluoride membranes, and detected by WesternBright ECL reagent (Advansta, USA). Primary anti-Flag and anti-β-actin antibodies from Sigma-Aldrich (USA), and secondary anti-mouse and anti-rabbit antibodies from GE Healthcare (USA) conjugated with horseradish peroxidase (HRP) have been used.

### Immunoprecipitation.

Human lung epithelial A549 cells and human embryonic kidney 293FT cells were transfected with Flag-tagged orf8 mammalian expression plasmids. At 48 h posttransfection, transfected cells were harvested in immunoprecipitation (IP) lysis buffer (20 mM Tris-Cl [pH 8.0], 150 mM NaCl, 0.5% NP-40) containing EDTA-free protease inhibitor cocktail (Roche) and clarified by centrifugation. Cell lysates were then immunoprecipitated using anti-Flag M2 magnetic beads (Millipore), followed by elution with 1× protein sample buffer with boiling. For immunoprecipitation from culture supernatant, the conditioned culture media were harvested and centrifuged at 1,000 × *g* for 20 min to remove cell debris. The clarified supernatants were then applied to equilibrated anti-Flag M2 magnetic beads for immunoprecipitation as described above.

### Liquid chromatography-mass spectrometry/mass spectrometry.

Serum samples of RT-qPCR-confirmed COVID-19 patients were handled in biosafety level 3 laboratory. Before heat inactivation, 14 abundant proteins, including albumin, IgG, IgA, IgM, IgD, IgE, fibrinogen, and transferrin, were removed by the High-Select Top14 abundant protein depletion spin column (Thermo Fisher Scientific). Depleted sera were mixed with final 1× protein sample buffer and denatured at 98°C for 10 min. Inactivated sera were loaded into 12% SDS-PAGE. The protein was stained with Coomassie blue and excised from the gel. The gel slice was then dehydrated with acetonitrile followed by overnight in-gel trypsin digestion. The peptides were then column cleaned using Pierce C_18_ spin columns (Thermo Fisher Scientific), followed by liquid chromatography coupled to tandem mass spectrometry (LC-MS/MS) analysis. MS data were further searched for matches to all amino acid sequences derived from SARS-CoV-2 (hCoV-19 WIV04, EPI_ISL_402124).

### Immunofluorescence staining.

A549 cells seeded on coverslips were transfected with pCAGEN-Flag-orf8. Cells were then fixed with 1:1 (vol/vol) acetone-methanol at −20°C, PBS washed, and stained with the indicated antibodies. Antibodies used for immunofluorescence staining were as follow: anti-Flag (Sigma-Aldrich), anti-calnexin (Abcam, UK), anti-α-tubulin (Abcam), anti-mouse labeled with Alexa Fluor 488 (Abcam), anti-rabbit labeled with Alexa Fluor 594 (Abcam). The coverslips were then mounted on glass microscope slides with Vectashield Vibrance mounting medium (VectorLabs, USA). Image acquisition was performed with a Zeiss LSM-880 confocal microscope with 63× oil objective (Zeiss). Z-stack acquisition was performed using ZEN software (Zeiss).

### RNA extraction, reverse transcription, and RT-PCR.

Total RNA was isolated from cultured cells using RNAiso Plus (TaKaRa, USA) following the manufacturer’s instructions. Complementary DNA (cDNA) was synthesized by reverse transcribing 1 μg purified RNA using PrimeScript RT reagent kit with gDNA Eraser (TaKaRa). RT-PCR was performed using DreamTaq Green DNA polymerase (Thermo Fisher Scientific).

### Multiple alignment and phylogenetic analysis.

Amino acid sequences of orf8 proteins are derived from bat SARS-like coronaviruses (bat SL-CoV WIV16, GenBank accession no. KT444582; bat SL-CoV WIV1, KF367457; bat SL-CoV Rs3367, KC881006; bat SL-CoV Rs672, FJ588686; bat SL-CoV RsSHC014, KC881005; bat SL-CoV Rp3, DQ071615; bat SARS-CoV HKU3-1, DQ022305; bat SARSr-CoV RaTG13, MN996532; bat SL-CoV ZXC21, MG772934; bat SL-CoV ZC45, MG772933; bat SL-CoV Rf1, DQ412042; bat SL-CoV YNLF-31C, KP886808; bat SL-CoV YNLF34C, KP886809), Paguma SARS-CoV (pSARS-CoV HC/SZ/61/03, AY515512), SARS-CoV (SARS-CoV GZ02, AY390556), and SARS-CoV-2 (hCoV-19 WIV04, EPI_ISL_402124; hCoV-19 WA2, PI_ISL_412970). Multiple alignment analysis was performed using on-line CLUSTALW software (www.genome.jp/tools-bin/clustalw), and the alignment of orf8 protein sequences was drawn using BoxShade program (embnet.vital-it.ch/software/BOX_form.html). Phylogenetic analysis of orf8 amino acid sequences was performed using MEGA X (24). After the multiple alignment and the calculation of the optimal parameter, the tree was built using neighbor-joining and JTT matrix-based method.

### Protein structure prediction.

SARS-CoV-2 orf8 protein structure prediction was done using comparative modeling with RosettaCM (25) on the Robetta protein structure prediction server. The *in silico* prediction was built based on the structures that were detected and aligned by HHSEARCH, SPARKS, and Raptor. The loop regions of the orf8 protein were assembled and optimized with reference to aligned structure templates. Different angles of the predicted structure were visualized using PyMOL (DeLano Scientific LLC). The topology was drawn based on the predicted structure.

### Abbott Architect testing.

Serum samples were run on the Abbott Architect instrument using the Abbott SARS-CoV-2 IgG assay following the manufacturer’s instructions. The assay is a chemiluminescent microparticle immunoassay for qualitative detection of IgG in human serum or plasma against the SARS-CoV-2 nucleoprotein. The Architect platform requires a minimum of 100 μl of serum or plasma. Qualitative results and index values reported by the instrument were used in analyses.

### ELISA.

Antigen-specific antibodies were examined by enzyme-linked immunosorbent assay (ELISA). Briefly, recombinant N protein purified from E. coli and recombinant orf8 protein purified from Expi293 cells were coated with 100 ng and 250 ng of protein per well in 50 mM coating buffer (pH 9.6 Na_2_CO_3_/NaHCO_3_) on ELISA plates (Maxisorp nuncimmuno plate, Thermo Fisher Scientific) and incubated overnight at 4°C. Plates were blocked with 0.5% (wt/vol) bovine serum albumin (BSA) and 0.5% gelatin in 0.05% Tween 20 (Sigma-Aldrich) in PBS at room temperature for 1 h and washed one time in 0.05% Tween 20 (Sigma) in PBS. Diluted sera were added and incubated for 1 h at 37°C. Plates were washed three times and incubated with HRP-conjugated goat anti-human IgM (Thermo Fisher Scientific, catalog no. A18841), goat anti-human IgG (Thermo Fisher Scientific, catalog no. A2290), or goat anti-human IgA (Thermo Fisher Scientific, catalog no. A18781) for 1 h at 37°C. The color was developed using trimethyl borane (TMB) solution (Thermo Fisher Scientific), and absorbance was measured at 450 nm using a Varioskan LUX multimode microplate reader (Thermo Fisher Scientific). We used archived anonymous serum samples as the negative control. The cutoff for seropositivity was set as the mean value of 100 control serum samples plus two times the standard deviation.

### Statistics.

All analysis was performed using GraphPad PRISM software. A paired or unpaired *t* test was used for analysis of two groups, and one-way analysis of variance (ANOVA) was used for analysis of multiple groups. Nonparametric data were analyzed by a Mann-Whitney test. *P* values of less than 0.05 were considered statistically significant. Data in this study were indicated as means with standard deviations.
