# Supplementary material for: Accurate Diagnosis of COVID-19 by a Novel Immunogenic Secreted SARS-CoV-2 orf8 Protein
Source: mBio. 2020 Oct 20;11(5):e02431-20. doi: 10.1128/mBio.02431-20 (PMC7587431; doi:10.1128/mBio.02431-20)
Supplement: TABLE S1 [file mBio.02431-20-st001.docx]

Table S1. Cohort characteristics of patients.

|  | Figure 4 | Figure 5 | Figure 6 |
| --- | --- | --- | --- |
| Number | 29 | 64 | 14 |
| Demographics | | | |
| Male (%) | 48% (14/29) | 50% (32/64) | 64% (9/14) |
| Female (%) | 52% (15/29) | 50% (32/64) | 36% (5/14) |
| Age (Years), Median (Range) | 56 (22-87) | 53 (20-87) | 70 (28-91) |
| Days Post Hospitalization at Collection (Days),  Median (Range) | 9 (0-29) | 2 (0-6) | 4 (0-8) |
| Days Post Symptom Onset at Collection (Days),  Median (Range) | 46 (28-62) | 4 (0-14) | 4 (0-8) |
| Disease Severity (%) |  |  |  |
| Asymptomatic | 0% (0/29) | 6% (4/64) | 29% (4/14) |
| Mild | 72% (21/29) | 74% (47/64) | 42% (6/14) |
| Severe | 28% (8/29) | 20% (13/64) | 29% (4/14) |
| Residency (%) |  |  |  |
| Queen Mary Hospital | 100% (29/29) | 100% (64/64) | 100% (14/14) |
| SARS-CoV-2 PCR Positivity | 100% (29/29 tested) | 100% (64/64 tested) | 100% (14/14 tested) |
| Oxygen supplementation | 28% (8/29) | 20% (13/64) | 29% (4/14) |
| ICU admission | 0% (0/29) | 3% (2/64) | 0% (0/14) |
